# An Adaptation of the Split-Cylinder Resonator Method for Measuring the Microwave Properties of Thin Ferroelectric Films in a “Thin Film—Substrate” Structure

**DOI:** 10.3390/s24030755

**Published:** 2024-01-24

**Authors:** Alexander Gagarin, Diana Tsyganova, Andrey Altynnikov, Andrey Komlev, Roman Platonov

**Affiliations:** Department of Physical Electronics and Technology, Saint Petersburg Electrotechnical University “LETI”, ul. Professora Popova 5, St. Petersburg 197022, Russia; diatsyganova@gmail.com (D.T.); agaltynnikov@etu.ru (A.A.); aekomlev@etu.ru (A.K.); raplatonov@etu.ru (R.P.)

**Keywords:** dielectric microwave parameters, permittivity, loss tangent, split-cylinder resonator, thin dielectric film, ferroelectric film

## Abstract

The split-cylinder resonator method was adapted to measure the microwave properties (dielectric permittivity and loss tangent) of thin ferroelectric films on a dielectric substrate. The mathematical model for calculating the resonance frequency of the split-cylinder resonator was adjusted for the “ferroelectric film—substrate” structure. An approach for correcting the gap effect based on calibrating with a single-layer dielectric was introduced and used to study two-layer dielectrics. The prototype of a split-cylinder resonator designed to measure single-layer dielectric plates at a frequency of 10 GHz was presented. The resonator calibration was performed using dielectric PTFE samples and fused silica, and an example of the correction function was suggested. The measurement error was estimated, and recommendations on the acceptable parameter range for the material under investigation were provided. The method was demonstrated to measure the microwave properties of a ferroelectric film on a fused silica substrate.

## 1. Introduction

For decades, using ferroelectric materials in thin-film form for device design at microwaves has been seen as a promising application. Despite the long history [1,2] and a number of fundamental flaws of ferroelectrics, their advantages continue to attract the attention of researchers to the subject [3,4,5,6]. The use of ferroelectrics, like any other dielectrics, requires an accurate measurement of their dielectric permittivity ε and dielectric losses tanδ or quality factor Q=1/tanδ . The main issues with these measurement objects are their high dielectric permittivity ε (ranging from hundreds to thousands), relatively high dielectric losses tanδ (around 0.01), and the impossibility of separating the thin ferroelectric film, which has a thickness in the μ m scale, from the dielectric substrate, with a relatively higher thickness (around 1 mm) and a dielectric permittivity of approximately 10.

The best way to measure the properties of thin ferroelectric films is by using planar [7,8,9,10,11] or sandwich [11,12,13] capacitors based on them. The properties of the ferroelectric film can be extracted from the overall capacitor parameters. The same principle applies to the manufacturing of slot lines and coplanar waveguides [14,15]. This principle is implemented by concentrating the electric field inside the thin film through the narrow gap between the capacitor plates. The disadvantage of that approach is, firstly, the need to intervene by creating a metal coating, and secondly, the uncertainties related to separating the metallization properties from the properties of the dielectric. Methods have been proposed [16,17] to overcome the latter problem, but there is a need for techniques to measure the microwave parameters of the dielectric film itself in its original state.

Since dielectric materials are commonly used in radio engineering, especially in microwave devices, various techniques have been developed to measure their microwave properties. These methods can be classified as the transmission/reflection line, open-ended coaxial probe, free space, and microwave resonance. Measurement equipment using these methods are standardized and commercially available. To measure the microwave parameters of solid dielectrics, one can use open-ended probe equipment such as SPEAG DAK (SPEAG, Zurich, Switherland) [18] and Keysight N1501A (Keysight, Santa Rosa, CA, USA) [19], as well as the resonant method equipment Keysight 85072A (Keysight, Santa Rosa, CA, USA) (split-cylinder resonator) [20]. However, the mentioned equipment is designed for use with single layer dielectrics and does not support the direct measurement of “thin film—substrate” structures.

Many methods for measuring the properties of thin ferroelectric films without using metal electrodes have been presented in the literature over the years. The most well-known methods for investigating dielectrics with microwaves were used to complete the task. The resonance methods are known for their accuracy and simplicity in measuring the microwave parameters of ferroelectric films, compared to other methods. The measurement methods developed earlier in the millimeter-wave range included the Fabry–Perot open resonator [21,22,23], ${\mathrm{TE}}_{10n}$ rectangular resonator [24] (used for thin films of ${\mathrm{TiO}}_{2}$ on a glass substrate), resonator based on partially filled [25,26,27,28] or cut-off [22] waveguides, and transmission between horns through a ferroelectric sample [27]. Additionally, measurements at frequencies around 10 GHz (a frequency standard for bulk dielectrics) were conducted using a composite dielectric resonator [29,30], microstrip resonator [7,31], and split-post dielectric resonator [28,32]. The most accurate methods are waveguide techniques, but they require the ferroelectric sample to be precisely cut to fit the waveguide cross-section perfectly. This operation is very difficult and expensive. Other techniques mentioned have lower accuracy but can be used directly with “thin film—substrate” samples, without any additional processing.

The split-cylinder resonator method also does not require additional processing. The equipment for this method is commercially available and widely used in material testing laboratories. The authors of this paper adapted the standard split-cylinder resonator method to measure the microwave parameters (dielectric permittivity and tangent loss) of thin dielectric films on a dielectric substrate. The mathematical model for calculating the resonance frequency of a split-cylinder resonator was modified by introducing a two-layered heterogeneous dielectric structure “thin film—substrate”. The model for a two-layered structure was experimentally validated by studying the microwave parameters of ferroelectric films deposited on a fused silica substrate.

## 2. Materials and Methods

### 2.1. Closed Resonator Model

There are some technical tasks that require a break in a regular waveguide. ${\mathrm{TE}}_{01}$ mode cylindrical waveguides are just a solution here because they have a zero longitudinal component of electric current and zero electric fields near the walls. An example of the implementation of the TE01 mode is a split-cylinder resonator used to measure dielectric parameters at microwave frequencies, as originally reported by Kent [33]. The initial solution for the resonator addressed a simplified problem for a closed-wall cylindrical waveguide. The solution was then adjusted using perturbation calculation to correct the error caused by the dielectric-filled gap [34]. The gap effect was also considered in full-wave analysis [35], studying the leakage of the electric field into the gap in more detail.

**Figure 1:**
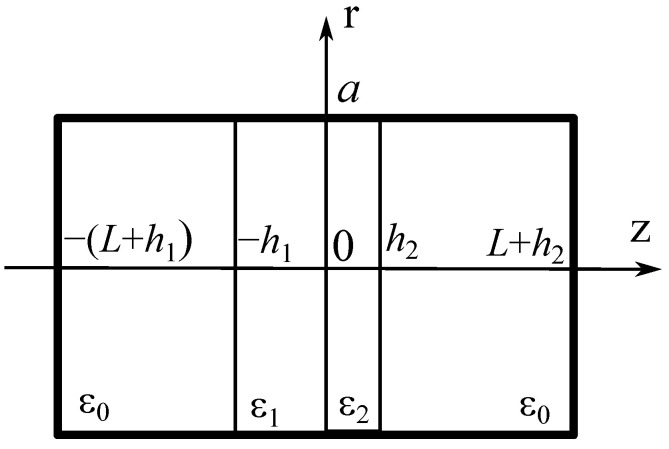
Schematic of the cylinder resonator with a two-layered dielectric.

The measurement of the parameters of a thin dielectric film on a substrate, placed into the gap of a split-cylinder resonator, can also be initially approached in a simplified way. Figure 1 shows a longitudinal schematic cross-section of a closed cylindrical resonator with a two-layered dielectric inclusion (*L*—length of each air-filled resonator section; $\varepsilon_{1}$ and $h_{1}$ —permittivity and thickness of the substrate, respectively; $\varepsilon_{2}$ and $h_{2}$ —permittivity and thickness of the thin film, respectively; and *a*—radius of the cylindrical waveguide).

For the ${\mathrm{TE}}_{01}$ mode, a cut-off wave number is defined as $\beta_{c}=\chi^{\prime }/a$ , where $\chi^{\prime }=3.832$ . The resonance condition for the structure presented in Figure 1 can be expressed as the following transcendental equation (see Appendix A.1)

(1) $$\begin{array}{c} \beta_{1}\left(1-\frac{\beta_{1}}{\beta_{0}}\tan\beta_{1}h_{1}\tan\beta_{0}L\right)\left(\tan\beta_{2}h_{2}+\frac{\beta_{2}}{\beta_{0}}\tan\beta_{0}L\right)+\\ \beta_{2}\left(1-\frac{\beta_{2}}{\beta_{0}}\tan\beta_{2}h_{2}\tan\beta_{0}L\right)\left(\tan\beta_{1}h_{1}+\frac{\beta_{1}}{\beta_{0}}\tan\beta_{0}L\right)=0, \end{array}$$

where

(2) $$\beta_{0}=\sqrt{{\left(\frac{2\pi f_{0}}{c}\right)}^{2}-{\left(\frac{\chi^{\prime }}{a}\right)}^{2}},$$

(3) $$\beta_{1}=\sqrt{\varepsilon_{1}{\left(\frac{2\pi f_{0}}{c}\right)}^{2}-{\left(\frac{\chi^{\prime }}{a}\right)}^{2}},$$

(4) $$\beta_{2}=\sqrt{\varepsilon_{2}{\left(\frac{2\pi f_{0}}{c}\right)}^{2}-{\left(\frac{\chi^{\prime }}{a}\right)}^{2}},$$

where $f_{0}$ is the resonance frequency and *c* is light velocity.

The numerical solution of Equation (1), together with Equations (2)–(4), allows us to find one variable (e.g., $f_{0}$ or $\varepsilon_{2}$ ) when the others are known. The solution for the frequency $f_{0}$ , if it exists, should be found within a range up to the resonance frequency $f_{e}$ of an empty cylindrical resonator ${\mathrm{TE}}_{011}$ of a $2L+h_{1}$ length (see Equation (5)).

(5) $$f_{e}=\frac{c}{2\pi }\sqrt{{\left(\frac{\pi }{2L+h_{1}}\right)}^{2}+{\left(\frac{\chi^{\prime }}{a}\right)}^{2}}.$$

### 2.2. The Gap Correction

The simplest and most reliable method of gap effect correction is calibration. The dielectric plate samples with known parameters (permittivity ε and thickness *h*) are placed into the resonator cavity, and the resonance frequencies are measured. The calculated results are then adjusted to match the experimental results using fitting parameters.

According to the full-wave analysis for a single-layered structure [35], the angular component of the electric field in the gap area rapidly decreases to a negligible value at a distance in a radial direction (along the *r*-axis) close to the sample thickness (the gap thickness) and is practically independent of the sample’s dielectric permittivity. The electric field in the rest of the gap area is perpendicular to the surface of the resonator flange (along the *z*-axis). Thus, if the thickness of the dielectric sample is much less than the width of the flange, we can consider the gap as a parallel-plate capacitor filled with this dielectric.

For a two-layer sample, the gap can be represented as a capacitor with the area *S* and the capacitance $C_{gap}$ , which is formed in series by the capacitance of the substrate $C_{sub}$ and the capacitance of the film $C_{film}$ as

$$\begin{array}{c} \frac{1}{C_{gap}}=\frac{1}{C_{sub}}+\frac{1}{C_{film}}=\frac{h_{1}}{\varepsilon_{0}\varepsilon_{1}S}+\frac{h_{2}}{\varepsilon_{0}\varepsilon_{2}S}=\frac{1}{\varepsilon_{0}S}\left(\frac{h_{1}}{\varepsilon_{1}}+\frac{h_{2}}{\varepsilon_{2}}\right). \end{array}$$

Taking into account that $\varepsilon_{1}<<\varepsilon_{2}$ and $h_{1}>>h_{2}$ , we can conclude that the gap capacitance is determined solely by the substrate parameters, and the gap correction parameter is independent of the film parameters. Thus, the calibration performed for the single-layer samples with the known parameters can also be used for measuring “ferroelectric film—substrate” samples.

The equation for a single-layered structure, which should be used during calibration, is derived by substituting $\beta_{1}=\beta_{2}=\beta$ and $h_{1}=h_{2}=h/2$ into Equation (1) (see Appendix A.2). The calculated results were adjusted to fit the experimental data obtained with calibration samples by introducing a correction function *P*, which resulted in the following equation:

(6) $$\begin{array}{c} 1-\frac{\beta }{\beta_{0}}\tan\beta \frac{h}{2}\tan\beta_{0}L=-P. \end{array}$$

The literature [34,35] indicates that the correction function *P* should depend on ε and *h*. The correction function *P*, obtained in the calibration procedure, is then used in the equation for a two-layered structure in the following way:

(7) $$\begin{array}{c} \beta_{1}\left(1-\frac{\beta_{1}}{\beta_{0}}\tan\beta_{1}h_{1}\tan\beta_{0}L+P\right)\left(\tan\beta_{2}h_{2}+\frac{\beta_{2}}{\beta_{0}}\tan\beta_{0}L\right)+\\ \beta_{2}\left(1-\frac{\beta_{2}}{\beta_{0}}\tan\beta_{2}h_{2}\tan\beta_{0}L+P\right)\left(\tan\beta_{1}h_{1}+\frac{\beta_{1}}{\beta_{0}}\tan\beta_{0}L\right)=0. \end{array}$$

### 2.3. Ferroelectric Film Microwave Parameter Measurements

The microwave parameters of the thin ferroelectric film on a dielectric substrate are measured by placing the sample between the halves of the split-cylinder resonator. The resonance for the two-layer sample should occur at a lower frequency than the resonance for the substrate only, which should be used for calibration. The directly measured parameters include resonance frequency $f_{0}$ , loaded Q-factor *Q*, and insertion loss IL at the resonance frequency. The measured parameters can then be used to extract the thin film parameters.

The film’s permittivity can be found by solving Equation (7) for $\varepsilon_{2}$ . The dielectric loss of the sample, tanδ , can be determined using the following expression

(8) $$\tan\delta =\frac{1}{\xi }\left(\frac{1}{Q_{0}}-\frac{1}{Q_{00}}\right),$$

where $Q_{00}$ is an unloaded Q-factor of the resonator with the substrate only, ξ is an inclusion coefficient (also known as filling factor), and $Q_{0}$ is an unloaded Q-factor of the resonator with the sample.

ξ is defined in the following [27,31]:

(9) $$\xi =-2\;\frac{\varepsilon_{2}}{f_{0}}\;\frac{df_{0}}{d\varepsilon_{2}},$$

where the derivative is numerically calculated for the resonance frequency measured $f_{0}$ by solving Equation (7).

The Unloaded Q-factor $Q_{0}$ is calculated from *Q* and IL . For the insertion loss IL defined in dB, $Q_{0}$ is expressed as

(10) $$Q_{0}=Q\;{\left(1-10^{-\frac{IL}{20}}\right)}^{-1}.$$

### 2.4. Measurement Error

The measurement errors of the film parameters ( ${\Delta \varepsilon }_{2}$ and Δtanδ ) were estimated using the measurement errors of the sample parameters ( ${\Delta \varepsilon }_{1}$ , ${\Delta h}_{1}$ , and ${\Delta h}_{2}$ ) and the measurement errors of the experimental values ( ${\Delta f}_{0}$ , Δ*Q*, and Δ IL ). The resonator parameters (*a*, *L*, and $Q_{00}$ ) have been excluded from the error estimation.

The measurement error of thin film permittivity ${\Delta \varepsilon }_{2}$ is defined by solving Equation (7) and is expressed as

(11) $${\Delta \varepsilon }_{2}=\left|\frac{\partial \varepsilon_{2}}{\partial f_{0}}\right|{\Delta f}_{0}+\left|\frac{\partial \varepsilon_{2}}{\partial {\Delta \varepsilon }_{1}}\right|\varepsilon_{1}+\left|\frac{\partial \varepsilon_{2}}{\partial h_{1}}\right|{\Delta h}_{1}+\left|\frac{\partial \varepsilon_{2}}{\partial h_{2}}\right|{\Delta h}_{2}.$$

The measurement error of thin film dielectric loss Δ tanδ is defined via Equations (8)–(10) as

(12) $$\begin{array}{c} \Delta \tan\delta =\left|\frac{\partial \tan\delta }{\partial f_{0}}\right|{\Delta f}_{0}+\left|\frac{\partial \tan\delta }{\partial \varepsilon_{1}}\right|{\Delta \varepsilon }_{1}+\left|\frac{\partial \tan\delta }{\partial h_{1}}\right|{\Delta h}_{1}+\\ \left|\frac{\partial \tan\delta }{\partial \varepsilon_{2}}\right|{\Delta \varepsilon }_{2}+\left|\frac{\partial \tan\delta }{\partial Q}\right|\Delta Q+\left|\frac{\partial \tan\delta }{\partial IL}\right|\Delta IL, \end{array}$$

where dependencies on $f_{0}$ , $\varepsilon_{1}$ , $h_{1}$ , and $\varepsilon_{2}$ are introduced via Equation (9), while ${\Delta \varepsilon }_{2}$ is calculated from Equation (11).

### 2.5. Dielectric Samples’ Preparation

To perform the calibration procedure, we used single-layer dielectric plates made of PTFE and fused silica with different thicknesses ( h=1.4,1.5,2 and 3 mm for PTFE, h=0.5 and 1 mm for silica).

The fused silica samples were manufactured by Elektrosteklo LLC (Moscow, Russia) with a permittivity of $\varepsilon_{1}=3.84$ and a thickness of $h_{1}$ = 0.5 and 1.0 mm.

PTFE samples were fabricated using cold pressing technology. PTFE powder was dried at a temperature of $\left(150\pm 10\right)$ °C to achieve a moisture of less than 0.02%. Then, a portion of the powder was weighed to obtain a given plate thickness, cooled to a temperature of −20 °C and mixed at 800 rpm for 20 min. After mixing, the portion was pressed in a mold on a hydraulic press at $\left(300\pm 25\right)$ kg/cm^2^ for 1 min. The produced plate was loaded into an oven with air circulation at a temperature of 200 °C, then the temperature was raised at a rate of 1 °C/min to 360 °C and held for 6 h, followed by slow cooling (less than 1 °C/min) to 200 °C. Finally, the sample was cooled at room temperature.

Two ferroelectric film samples with a composition of Ba_0.3_Sr_0.7_TiO_3_ (sample 2) and SrTiO_3_ (sample 3) were deposited using the Tokuda CFS-4ES sputter system. The deposition was performed on a fused silica substrate with a thickness of 0.5 mm and a temperature of 400 °C at a pressure of working gas (85% Ar–15% O_2_) of 15 mTorr at a power of 300 W for 240 min. The samples were post-annealed in air at a temperature of 1000 °C (heating time 120 min, holding time 120 min).

The permittivity of the one-layer samples (PTFE and silica) was measured using parallel-plate capacitor measurements at a frequency of 1 MHz with GW Instek LCR-78201, (GW Instek, Taipei, China) assuming that the permittivity had the same value at microwaves ( ε=2.1 for PTFE, ε=3.84 for silica).

### 2.6. Measuring Resonator Design and Measurement Procedure

**Figure 2:**
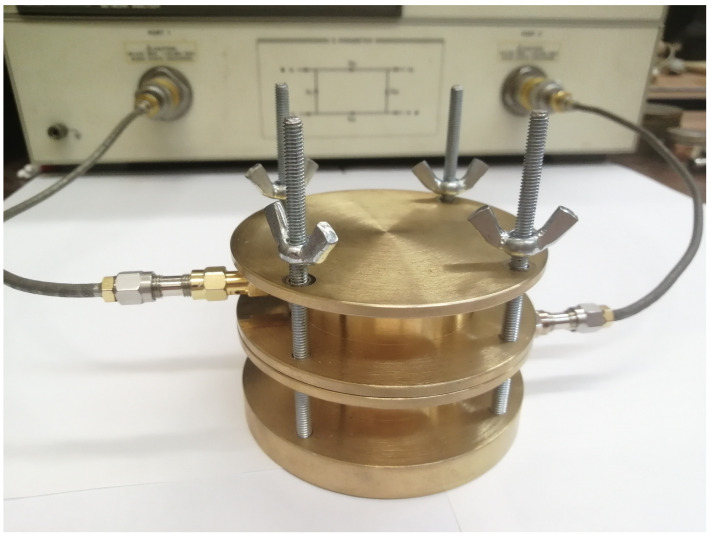
The overview of the split-cylinder resonator.

The split-cylinder resonator was designed to measure the parameters of dielectric plates at a frequency of 10 GHz. In accordance with Kent’s recommendations [33], the resonator parameters were chosen as follows: a cavity radius of a=20 mm and a resonator length of 2L=32 mm. The resonator halves were aligned using four threaded pins that passed through holes in four flanges with a radius of 80 mm (twice the radius of the cavity) on the inner and outer sides of the resonator halves. The $TE_{011}$ e was excited by coupling loops that terminate the feeding coaxial line inside the resonator. Figure 2 shows an overview of the resonator.

**Figure 3:**
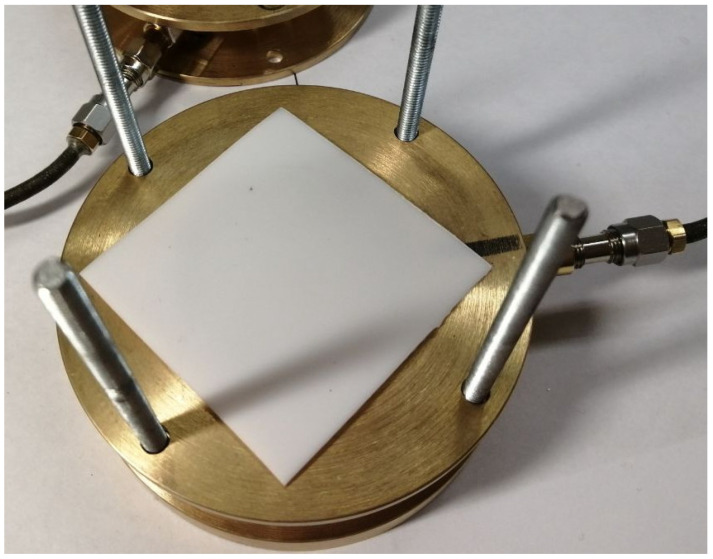
The PTFE sample placed for the measurement procedure.

**Figure 4:**
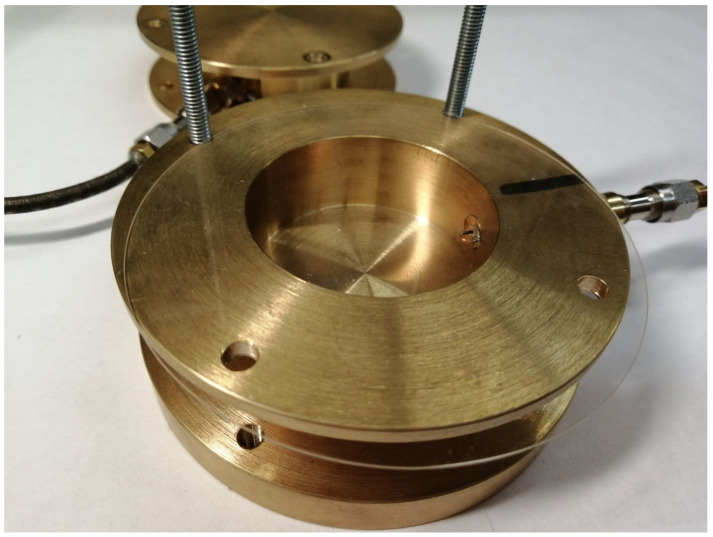
The ferroelectric film on the silica substrate sample placed for the measurement procedure (the coupling loop is visible inside the resonator cavity).

Dielectric samples are installed between the resonator halves. After separating the resonator halves, a dielectric sample is placed on the lower half. Figure 3 shows a PTFE sample positioned for measurement. The resonator’s cavity must be entirely covered with the sample and have an overlap on the flanges that is at least twice the thickness of the dielectric. Figure 4 shows a silica substrate with a deposited ferroelectric film. It is evident that up to two aligning pins can be removed to install the sample.

**Figure 5:**
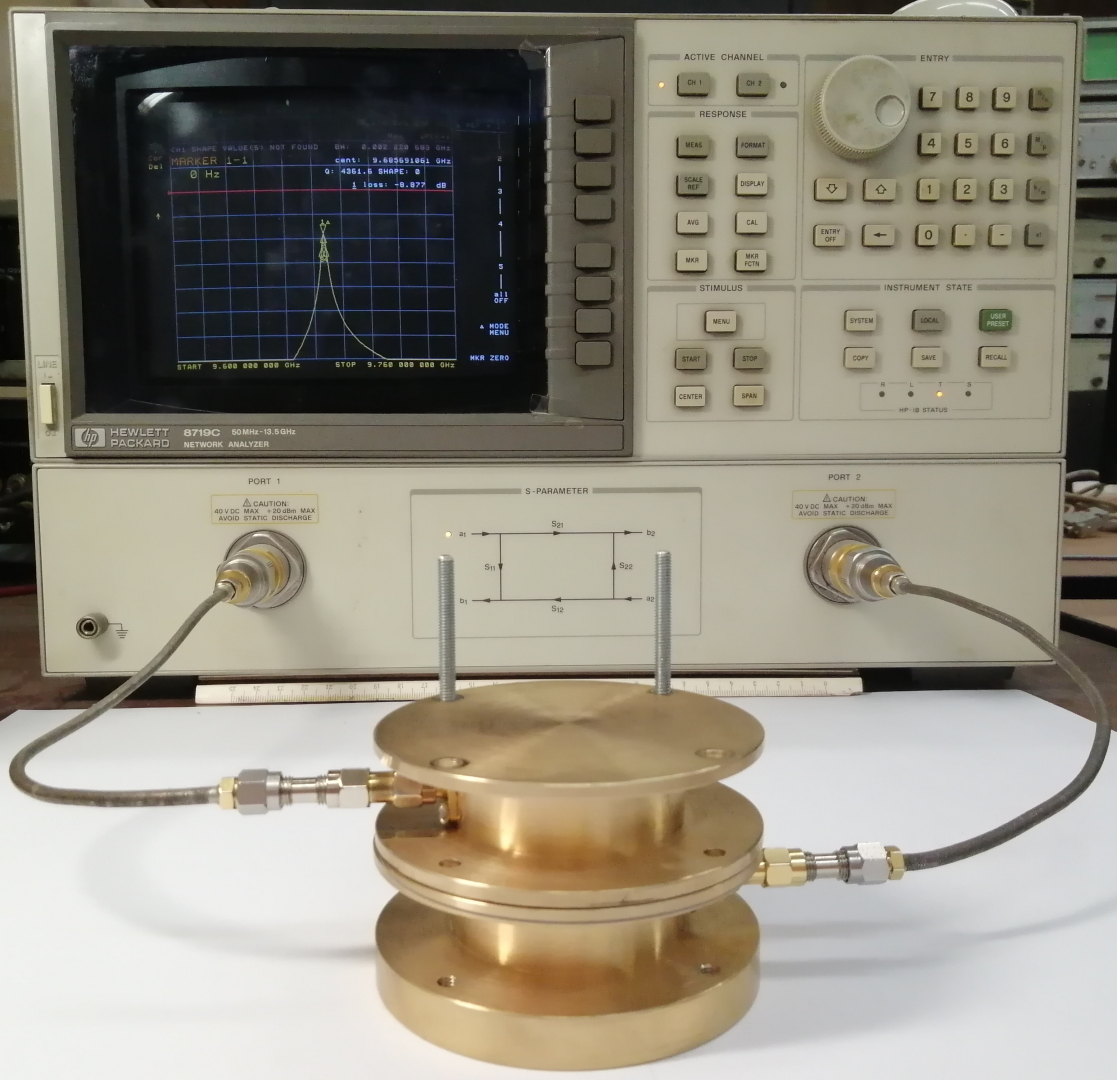
The measurement procedure using network analyzer HP 8719C.

To measure the resonator’s frequency response, it is connected to the HP 8719C network analyzer (Hewlett Packard Co., Santa Rosa, CA, USA) in transmission coefficient measurement mode ( $S_{21}$ -mode). Figure 5 illustrates the measurement procedure with the fused silica sample installed in the resonator.

## 3. Results

### 3.1. The Resonator Calibration

**Figure 6:**
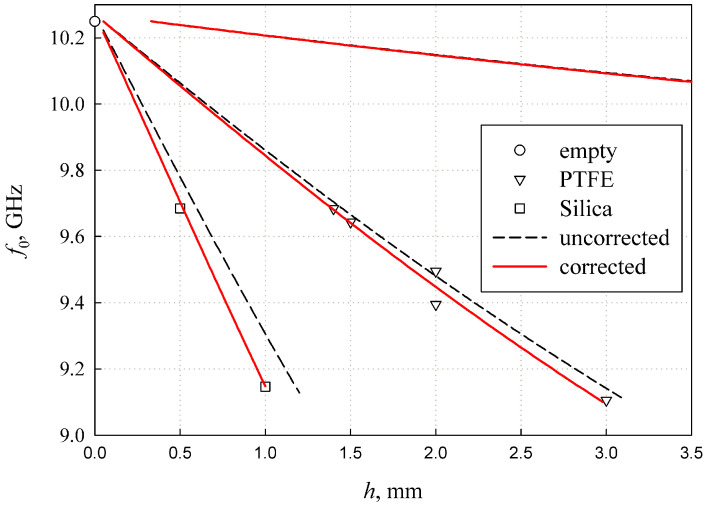
Results of the calibration (dashed lines for uncorrected calculations, solid lines for calculations corrected by parameter *P*).

Figure 6 shows the results of resonance frequency measurements for different reference samples used for calibration. Dashed lines show the calculation results for the initial Equation (6) without any correction, i.e., when P=0 . Calibration was performed based on the following correction function:

(13) $$P\left(\varepsilon ,h\right)=A{\left(\varepsilon -B\right)}^{2}\left(\frac{h}{2L}\right)\frac{\tan\beta_{0}L}{\beta_{0}L},$$

where A=0.7 and B=1.2 .

The specific format of the correction parameter function is not crucial, as long as it produces calculations that align with the experimental data using reference samples.

### 3.2. Resonance Frequency of Two-Layer Structure with the Ferroelectric Film

As described in Section 3.1, Equation (7) was used to calculate the resonance characteristics by substituting the correction function $P\left(\varepsilon ,h\right)$ (refer to Equation (13)).

**Figure 7:**
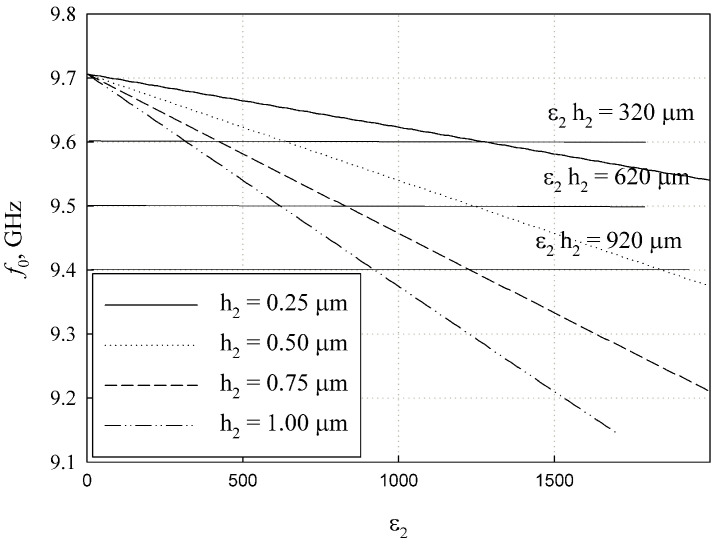
Resonance frequency versus film permittivity $\varepsilon_{2}$ at the different values of film thickness $h_{2}$ .

Figure 7 illustrates the variation in resonance frequency values with the film permittivity $\varepsilon_{2}$ for different film thicknesses $h_{2}$ . The calculation was performed for a silica substrate with $\varepsilon_{1}=3.84$ and $h_{1}=0.5$ mm, which was used in the calibration procedure. It can be seen that the value determining the resonance frequency of the structure is actually a product $\varepsilon_{2}h_{2}$ .

**Figure 8:**
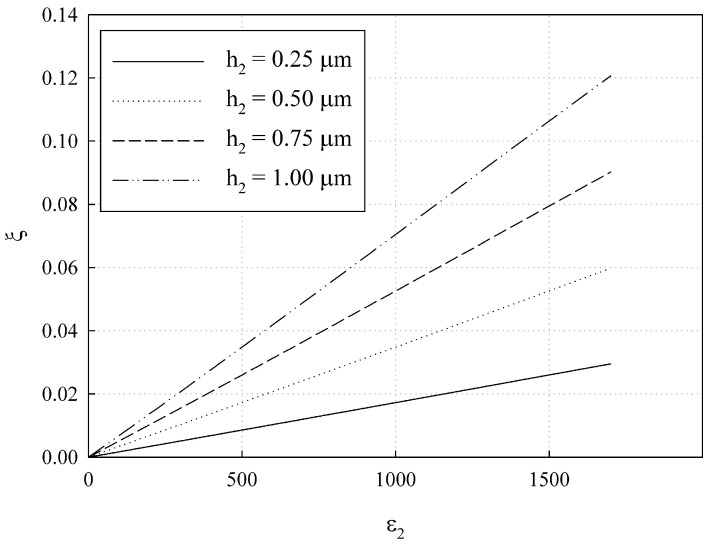
Inclusion coefficient ξ versus film permittivity $\varepsilon_{2}$ at the different values of film thickness $h_{2}$ .

Figure 8 presents the inclusion coefficient ξ versus the film permittivity $\varepsilon_{2}$ for the same substrate at a resonance frequency of $f_{0}=9.5$ GHz. It can be observed that the product $\varepsilon_{2}h_{2}$ defines ξ in the same way as mentioned earlier.

### 3.3. Estimation of Measurement Error

Measurement error for the thin film permittivity was calculated by Equation (11) for the substrate parameters $\varepsilon_{1}=3.84$ and $h_{1}=0.5$ mm, thin film parameters $\varepsilon_{2}=500$ and $h_{2}=0.5\;\mu$ m, and resonance frequency $f_{0}=9.5$ GHz.

**Figure 9:**
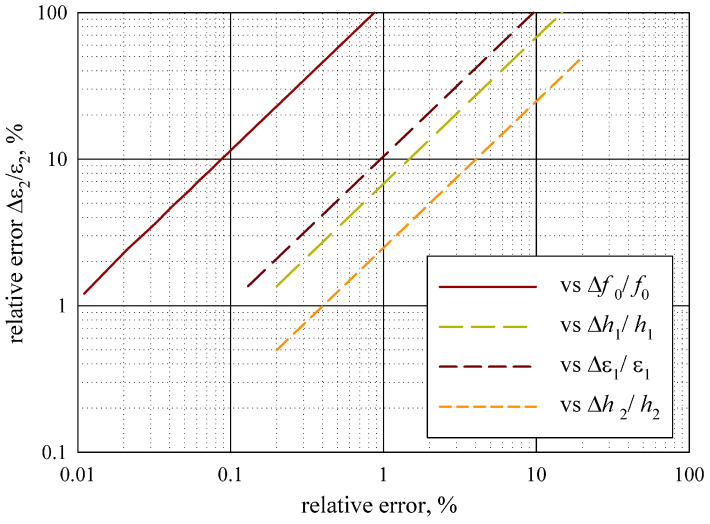
Influence of the specific parameters’ measurement errors.

At first, we estimated the influence of the measurement error of different parameters, as shown in Figure 9.

**Figure 10:**
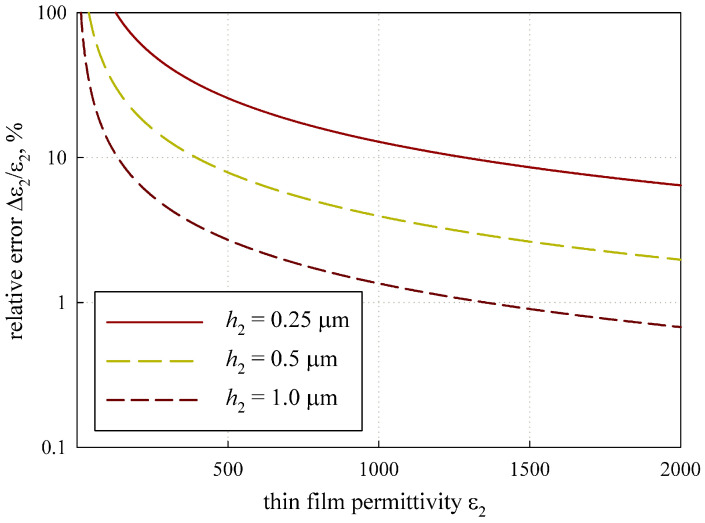
Influence of the thin film parameters on measurement error.

The resonance frequency error seems to be the most critical issue. However, The influence of this factor is moderated by the high Q-factor of the resonance and the use of modern microwave measurement setups, ensuring that the resonance frequency error is no more than 0.01% (or not more than ${\Delta f}_{0}=1$ MHz at $f_{0}=10$ GHz). If the remaining parameters are measured with errors of no more than 0.1%, then the overall error will not surpass 5%. These values were used to estimate how much the total measurement error ${\Delta \varepsilon }_{2}$ depends on the permittivity and thickness of the thin film (see Figure 10).

It can be observed from Figure 10 that the measurement error sharply increases for thin films with a permittivity of less than $\varepsilon_{2}=$ 500, while thicker films can be measured more accurately.

**Figure 11:**
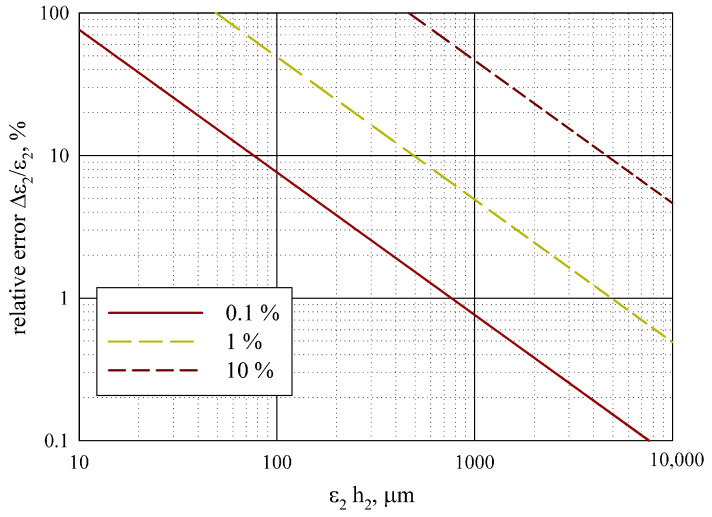
Measurement error versus product $\varepsilon_{2}h_{2}$ for Δ $f_{0}/f_{0}=0.01$ % at other parameters’ errors.

The measurement error Δ $\varepsilon_{2}$ can also be estimated depending on $\varepsilon_{2}h_{2}$ . Figure 11 displays the relative measurement error Δ $\varepsilon_{2}/\varepsilon_{2}$ for different values of Δ $\varepsilon_{1}/\varepsilon_{1}$ , Δ $h_{1}/h_{1}$ and Δ $h_{2}/h_{2}$ , all set to be equal, with Δ $f_{0}/f_{0}=0.01$ %.

### 3.4. Experimental Results on the Measurement of the Thin Ferroelectric Film

The Ba_0.3_Sr_0.7_TiO_3_ (sample 2) and SrTiO_3_ (sample 3) ferroelectric films were analyzed using the split-cylinder resonator. The pure fused silica substrate (sample 1) with the same thickness $h_{1}=0.5$ mm as the ferroelectric samples was also studied to determine the unloaded Q-factor $Q_{00}$ .

**Figure 12:**
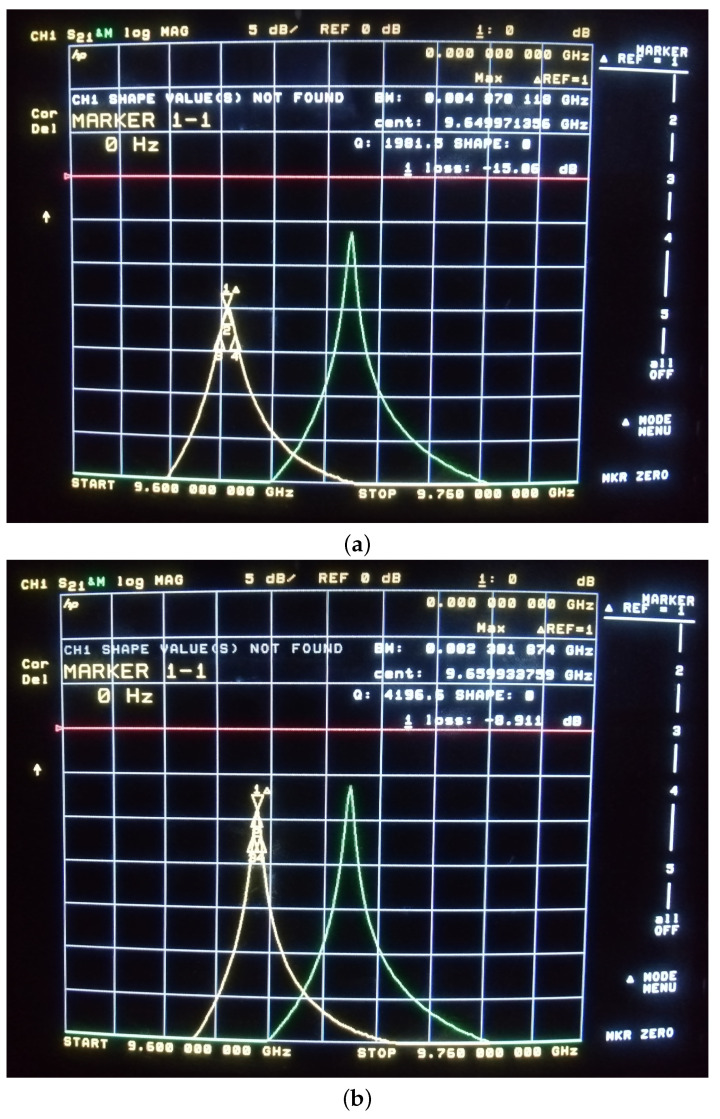
The view of the network analyzer display connected to the split-cylinder resonator with Sample 2 (**a**) and Sample 3 (**b**) installed.

Figure 12 shows the photograph of the network analyzer display while the split-cylinder resonator with samples 2 and 3 is connected. The right-positioned resonance peak is the memorized frequency response of the resonator with sample 1 (the pure fused silica substrate).

**Figure 13:**
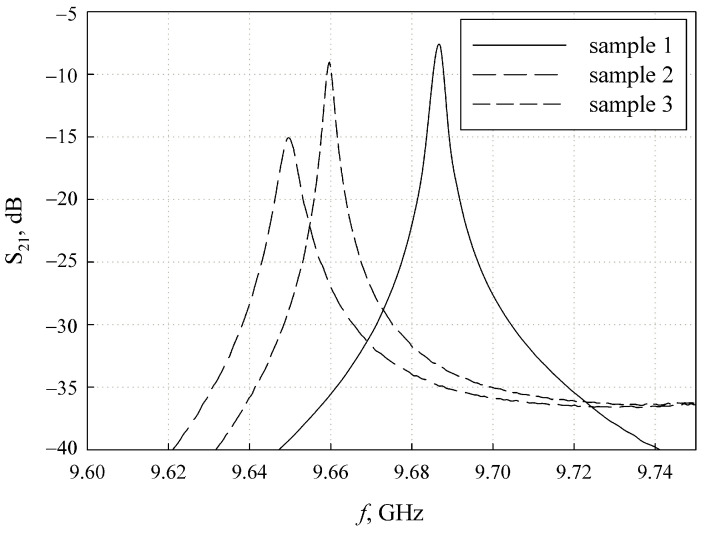
The experimental resonance curves of the split-cylinder resonator with the different dielectric samples.

The same results downloaded from the network analyzer are also shown in Figure 13.

The measured parameters and the calculated parameters using Equations (7)–(12) for the dielectric samples studied are presented in Table 1. The measurement error was calculated for Δ $f_{0}=1$ MHz, Δ $h_{1}=1\;\mu$ m, Δ $\varepsilon_{1}=0.01$ , and Δ $h_{2}=10$ nm.

## 4. Discussion

The proposed method allows for measuring the parameters of thin ferroelectric films on a substrate using equipment originally designed for measuring microwave parameters of homogeneous dielectric plates with a thickness on a millimeter scale. It can be a commercially available material-testing equipment, such as the split-cylinder resonator Keysight 85072A [20], or a custom-made split-cylinder resonator. The method utilizes the gap correction function *P*, which is obtained via calibration for a single-layer dielectric.

The analysis conducted in Section 3.2 and presented in Figure 7 shows that the ferroelectric film induces a measurable shift in the resonance frequency of the $TE_{011}$ -mode. The high resonance q-factor allows us to distinguish between the resonance with and without the ferroelectric film, despite the relatively low inclusion factor ξ (see Figure 8).

The common issue with thin films is the difficulty in accurately measuring their thickness. The current method shows that the key resonance parameters depend on the product $\varepsilon_{2}h_{2}$ (see Figure 7 and Figure 8). This implies that the measurement results ( $\varepsilon_{2}$ and tanδ ) are valid for a range of values of $\varepsilon_{2}h_{2}$ , rather than specific values of $h_{2}$ , helping to reduce uncertainty in film thickness measurement. This is because $\varepsilon_{2}$ and tanδ are determined in linear proportion to $h_{2}$ .

The analysis of the measurement error in Section 3.3 demonstrates that the method provides a permittivity measurement error Δ $\varepsilon_{2}/\varepsilon_{2}<10$ % for thin dielectric films with $\varepsilon_{2}h_{2}>100$ μ m, given that the errors for $\varepsilon_{1}$ , $h_{1}$ , and $h_{2}$ are less than 0.1%, and for films with $\varepsilon_{2}h_{2}>500$ μ m, if the errors are less than 1%. These limitations generally correspond to the ferroelectric films and allow for measuring them.

To verify the results of the measurement of the ferroelectric film parameters, we fabricated sets of planar capacitors using the ferroelectric samples as a base. Then, the capacitors were measured using the suspended stripline resonator (see Appendix B). The results of the capacitor parameters’ measurement were processed to extract the parameters of the ferroelectric films ( ε=329±4 and tanδ=0.026±0.003 for Sample 2; ε=267±5 and tanδ=0.004±0.0002 for Sample 3). Thus, the results obtained by the measurements with the split-cylinder resonator and with the planar capacitors differ by about 4%.

The proposed method was compared with earlier developed methods for measuring the microwave parameters of ferroelectric film without depositing metal electrodes in Table 2. The measurement errors are provided as claimed by the authors of the referenced works.

The results from our comparison suggest that the proposed method has lower accuracy compared to methods specifically developed for measuring ferroelectric films. However, the method proposed in this paper does not require specially designed test-fixtures, but it uses a standard one (split-cylinder resonator). Thus, the accuracy of the method presented can be considered good enough for preliminary measurements.

## 5. Conclusions

Our study suggests using the split-cylinder resonator method, a commonly used technique in microwave testing of dielectric materials, to evaluate the microwave properties of thin ferroelectric films on a dielectric substrate. Laboratories that test the microwave properties of dielectrics can use this method with the standard testing installations.

The permittivity and dielectric loss of the ferroelectric film can be extracted from the resonance characteristics by numerically solving the transcendental equation. By analyzing the measurement error, it was shown that the technique has an accuracy rate of approximately 10% for measuring the permittivity and loss tangent of ferroelectric films, if the product of the film permittivity $\varepsilon_{2}$ on the film thickness $h_{2}$ is more than 100 μ m, but rapidly decreases for the films with $\varepsilon_{2}h_{2}<100$ μ m.

The experiment with ferroelectric film on the silica substrate was performed to verify the possibility of method implementation. The permittivity and loss tangent of Ba_0.3_Sr_0.7_TiO_3_ and SrTiO_3_ ferroelectric films with a thickness of 0.5 μ m were analyzed using the method proposed. The film parameters were verified by the earlier developed measurement technique and fit with the results of measurements by the method proposed within a 4% difference.

Note that the method described in the present paper can be used not only for ferroelectric films’ parameters measurements, but for the measurements of any “thin dielectric film—substrate” two-layer structures, as long as the product of the film permittivity on the film thickness meets the method requirements.

## Figures and Tables

**Table 1:** Experimental and calculated results for the dielectric samples measurements.

Sample	$f_{0}$ , GHz	*Q*	IL , dB	$Q_{0}$	$h_{2}$ , μ m	$\varepsilon_{2}$	ξ	tanδ
1	9.687	4300	7.6	7500	-	-	-	-
2	9.650	1990	15.9	2370	0.5	340±30	0.012	0.025±0.003
3	9.660	4100	9.1	6050	0.5	278±30	0.009	0.0033±0.0005

**Table 2:** Comparison of the measurement methods.

Measurement Method	Measurement Frequency, GHz	Permittivity Measurement Error $\frac{\Delta \varepsilon }{\varepsilon }$ , %	Dielectric Loss Measurement Error $\frac{\Delta \tan\delta }{\tan\delta }$ , %	References
DR *	6	30–60	30–60	[29]
OR **	50	5	10	[23]
OR **	40	15	15	[21]
PFWG ***	10	5	10	[26]
PFWG ***	30	5	5	[27]
SPR ****	19	2–5	3–6	[32]
CR *****	10	2.5	4	[24]
SCR ******	10	9.6	12.7	this work

* DR—dielectric resonator; ** OR—open Fabry–Perot resonator; *** PFWG—partially filled waveguide; **** SPR—split-post resonator; ***** CR—rectangular cavity resonator; ****** SCR—split-cylinder resonator.

**Figure A1:**
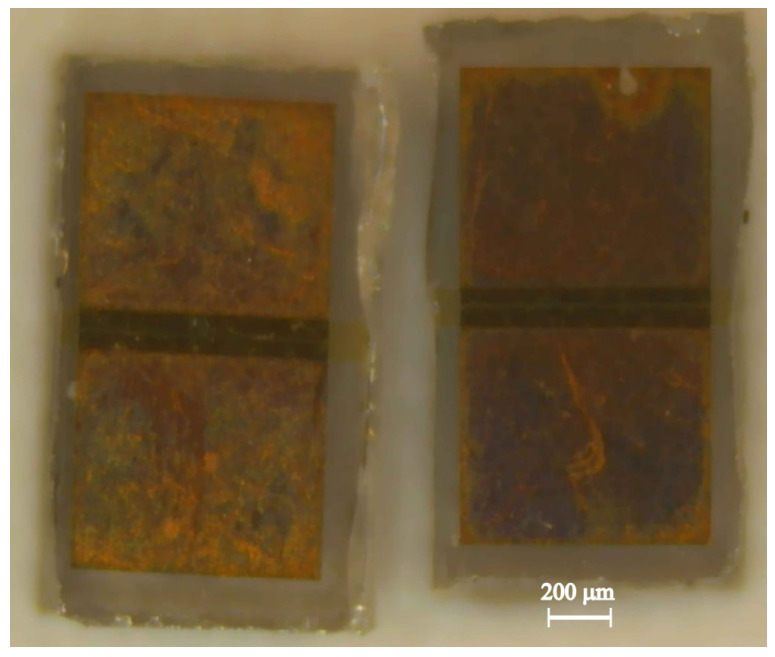
Planar capacitors.

**Figure A2:**
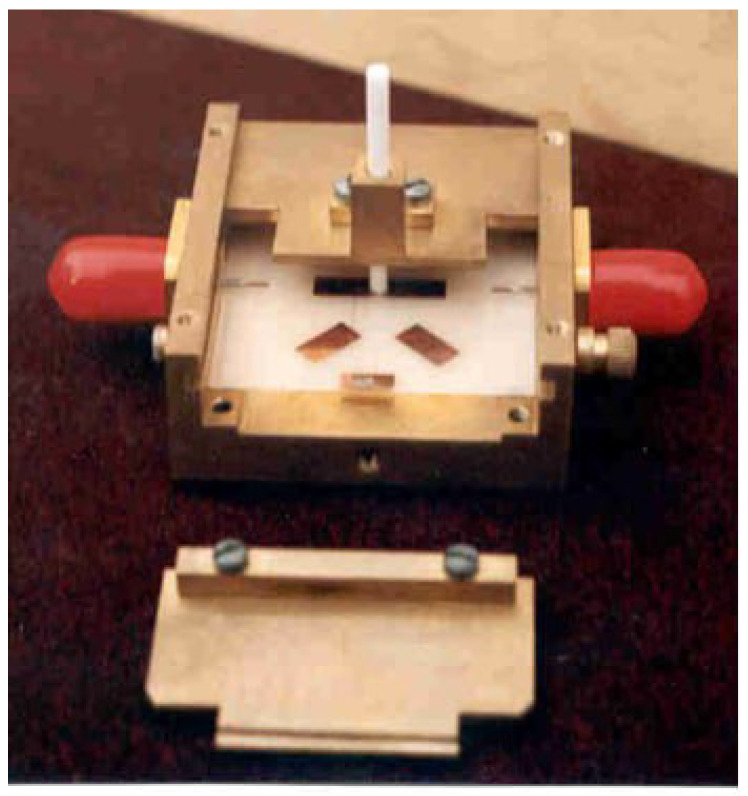
Suspended stripline 10 GHz resonator.

## Data Availability

The original contributions presented in the study are included in the article, further inquiries can be directed to the corresponding author.

## Appendix A. Derivation of the Dispersion Equation

### Appendix A.1. The Equation for the Two-Layer Structure

The resonance conditions are calculated for the ${\mathrm{TE}}_{011}$ mode in the cylindrical resonator presented in Figure 1. The conditions can be derived from the continuity and smoothness of the longitudinal component of the magnetic field at the dielectric layers interfaces together with the conditions on the conducting wall at the resonator short-circuited ends.

For the four different resonator sections, the longitudinal components of the magnetic field are expressed as follows

$$H_{z1}\left(z\right)=A\sin\beta_{0}\left(z+(L+h_{1})\right),$$

$$H_{z2}\left(z\right)=B\sin\beta_{1}z+C\cos\beta_{1}z,$$

$$H_{z3}\left(z\right)=D\sin\beta_{2}z+E\cos\beta_{2}z,$$

$$H_{z4}\left(z\right)=-F\sin\beta_{0}\left(z-(L+h_{2})\right).$$

The first derivatives of the components are

$$H_{z1}^{\prime }\left(z\right)=A\beta_{0}\cos\beta_{0}\left(z+(L+h_{1})\right),$$

$$H_{z2}^{\prime }\left(z\right)=B\beta_{1}\cos\beta_{1}z-C\beta_{1}\sin\beta_{1}z,$$

$$H_{z3}^{\prime }\left(z\right)=D\beta_{2}\cos\beta_{2}z-E\beta_{2}\sin\beta_{2}z,$$

$$H_{z4}^{\prime }\left(z\right)=-F\beta_{0}\cos\beta_{0}\left(z-(L+h_{2})\right).$$

So, the conditions on three interfaces $z=-h_{1}$ , z=0 , and $z=h_{2}$ give the system of equations for the amplitude coefficients

$$A\sin\beta_{0}L=-B\sin\beta_{1}h_{1}+C\cos\beta_{1}h_{1},$$

$$A\beta_{0}\cos\beta_{0}L=B\beta_{1}\cos\beta_{1}h_{1}+C\beta_{1}\sin\beta_{1}h_{1},$$

C=E,

$$B\beta_{1}=D\beta_{2},$$

$$D\sin\beta_{2}h_{2}+E\cos\beta_{2}h_{2}=F\sin\beta_{0}L,$$

$$D\beta_{2}\cos\beta_{2}h_{2}-E\beta_{2}\sin\beta_{2}h_{2}=-F\beta_{0}\cos\beta_{0}L.$$

Non-zero solutions of the system exist when

$$\det\left[\begin{array}{cccccc} \sin\beta_{0}L & \sin\beta_{1}h_{1} & -\cos\beta_{1}h_{1} & 0 & 0 & 0\\ \beta_{0}\cos\beta_{0}L & -\beta_{1}\cos\beta_{1}h_{1} & -\beta_{1}\sin\beta_{1}h_{1} & 0 & 0 & 0\\ 0 & 0 & 1 & 0 & -1 & 0\\ 0 & \beta_{1} & 0 & -\beta_{2} & 0 & 0\\ 0 & 0 & 0 & \sin\beta_{2}h_{2} & \cos\beta_{2}h_{2} & -\sin\beta_{0}L\\ 0 & 0 & 0 & \beta_{2}\cos\beta_{2}h_{2} & -\beta_{2}\sin\beta_{2}h_{2} & \beta_{0}\cos\beta_{0}L \end{array}\right]=0,$$

(A1)
$$\begin{array}{c} \beta_{0}^{2}\beta_{1}\cos\beta_{1}h_{1}\sin\beta_{2}h_{2}\cos^{2}\beta_{0}L+\beta_{0}^{2}\beta_{2}\sin\beta_{1}h_{1}\cos\beta_{2}h_{2}\cos^{2}\beta_{0}L\\ -\beta_{0}\beta_{1}^{2}\sin\beta_{1}h_{1}\sin\beta_{2}h_{2}\cos\beta_{0}L\sin\beta_{0}L-\beta_{0}\beta_{2}^{2}\sin\beta_{1}h_{1}\sin\beta_{2}h_{2}\cos\beta_{0}L\sin\beta_{0}L\\ -\beta_{1}^{2}\beta_{2}\sin\beta_{1}h_{1}\cos\beta_{2}h_{2}\sin^{2}\beta_{0}L-\beta_{1}\beta_{2}^{2}\cos\beta_{1}h_{1}\sin\beta_{2}h_{2}\sin^{2}\beta_{0}L\\ +2\beta_{0}\beta_{1}\beta_{2}\cos\beta_{1}h_{1}\cos\beta_{2}h_{2}\sin\beta_{0}L\cos\beta_{0}L=0 \end{array}$$

Dividing Equation (A1) by $\cos\beta_{1}h_{1}\cos\beta_{2}h_{2}\cos^{2}\beta_{0}L$ gives

$$\begin{array}{c} \beta_{0}^{2}\beta_{1}\tan\beta_{2}h_{2}+\beta_{0}^{2}\beta_{2}\tan\beta_{1}h_{1}\\ -\beta_{0}\beta_{1}^{2}\tan\beta_{1}h_{1}\tan\beta_{2}h_{2}\tan\beta_{0}L-\beta_{0}\beta_{2}^{2}\tan\beta_{1}h_{1}\tan\beta_{2}h_{2}\tan\beta_{0}L\\ -\beta_{1}^{2}\beta_{2}\tan\beta_{1}h_{1}\tan^{2}\beta_{0}L-\beta_{1}\beta_{2}^{2}\tan\beta_{2}h_{2}\tan^{2}\beta_{0}L\\ +\beta_{0}\beta_{1}\beta_{2}\tan\beta_{0}L+\beta_{0}\beta_{1}\beta_{2}\tan\beta_{0}L=0, \end{array}$$

$$\begin{array}{c} \beta_{1}\left[\beta_{0}^{2}\tan\beta_{2}h_{2}-\beta_{0}\beta_{1}\tan\beta_{1}h_{1}\tan\beta_{2}h_{2}\tan\beta_{0}L-\beta_{1}\beta_{2}\tan\beta_{1}h_{1}\tan^{2}\beta_{0}L+\beta_{0}\beta_{2}\tan\beta_{0}L\right]+\\ \beta_{2}\left[\beta_{0}^{2}\tan\beta_{1}h_{1}-\beta_{0}\beta_{2}\tan\beta_{1}h_{1}\tan\beta_{2}h_{2}\tan\beta_{0}L-\beta_{1}\beta_{2}\tan\beta_{2}h_{2}\tan^{2}\beta_{0}L+\beta_{0}\beta_{1}\tan\beta_{0}L\right]=0, \end{array}$$

$$\begin{array}{c} \beta_{1}\left[\beta_{0}^{2}\tan\beta_{2}h_{2}\left(1-\frac{\beta_{1}}{\beta_{0}}\tan\beta_{1}h_{1}\tan\beta_{0}L\right)+\beta_{0}\beta_{2}\tan\beta_{0}L\left(1-\frac{\beta_{1}}{\beta_{0}}\tan\beta_{1}h_{1}\tan\beta_{0}L\right)\right]+\\ \beta_{2}\left[\beta_{0}^{2}\tan\beta_{1}h_{1}\left(1-\frac{\beta_{2}}{\beta_{0}}\tan\beta_{2}h_{2}\tan\beta_{0}L\right)+\beta_{0}\beta_{1}\tan\beta_{0}L\left(1-\frac{\beta_{2}}{\beta_{0}}\tan\beta_{2}h_{2}\tan\beta_{0}L\right)\right]=0, \end{array}$$

(A2)
$$\begin{array}{c} \beta_{1}\beta_{0}^{2}\left[\left(1-\frac{\beta_{1}}{\beta_{0}}\tan\beta_{1}h_{1}\tan\beta_{0}L\right)\left(\tan\beta_{2}h_{2}+\frac{\beta_{2}}{\beta_{0}}\tan\beta_{0}L\right)\right]+\\ \beta_{2}\beta_{0}^{2}\left[\left(1-\frac{\beta_{2}}{\beta_{0}}\tan\beta_{2}h_{2}\tan\beta_{0}L\right)\left(\tan\beta_{1}h_{1}+\frac{\beta_{1}}{\beta_{0}}\tan\beta_{0}L\right)\right]=0. \end{array}$$

Dividing Equation (A2) by $\beta_{0}^{2}$ finally gives

(A3)
$$\begin{array}{c} \beta_{1}\left(1-\frac{\beta_{1}}{\beta_{0}}\tan\beta_{1}h_{1}\tan\beta_{0}L\right)\left(\tan\beta_{2}h_{2}+\frac{\beta_{2}}{\beta_{0}}\tan\beta_{0}L\right)+\\ \beta_{2}\left(1-\frac{\beta_{2}}{\beta_{0}}\tan\beta_{2}h_{2}\tan\beta_{0}L\right)\left(\tan\beta_{1}h_{1}+\frac{\beta_{1}}{\beta_{0}}\tan\beta_{0}L\right)=0. \end{array}$$

Numerically solving Equation (A3) for resonance frequency $f_{0}$ in a range from the cut-off frequency to the resonance frequency of the empty ${\mathrm{TE}}_{011}$ cylinder resonator of the length 2L gives a single root. In the same way, the equation can be solved for the dielectric layer permittivity.

### Appendix A.2. Reducing the Equation for the Single-Layer Structure

In the case of a single dielectric layer with $\beta_{1}=\beta_{2}=\beta$ and $h_{1}=h_{2}=h/2$ , Equation (A3) is reduced to the system of transcendental equations

(A4)
$$1-\frac{\beta }{\beta_{0}}\tan\beta \frac{h}{2}\tan\beta_{0}L=0,$$

(A5)
$$\tan\beta \frac{h}{2}+\frac{\beta }{\beta_{0}}\tan\beta_{0}L=0.$$

Equation (A5) gives false roots on its own, while Equation (A4), using the substitution with Kent’s [33] notation θ=βh/2 and $\psi =\beta_{0}L$ , exactly gives Kent’s Equation [33]

(A6)
$$\theta \tan\theta =\frac{h}{2L}\psi \cot\psi .$$

## Appendix B. Thin Ferroelectric Film Parameters’ Verification

For the verification of the obtained measurement results of ferroelectric film parameters (dielectric permittivity and loss tangent), planar capacitors were manufactured on the base of the investigated “thin film—substrate” structure.

In order to manufacture the capacitors, the samples’ surface was covered with a metal film with a thickness of approximately 1 μ m. The metal film initially went through a photolithography process for the formation of a set of planar capacitors with the following topological parameters: the gap thickness of 5 μ m, the gap length of 800 μ m, and the capacitor length (the size in the direction orthogonal to the capacitor gap) of 1600 μ m. Appearance of typical manufactured capacitors is shown in Figure A1.

The manufactured capacitors were investigated by means of measuring the resonator with a suspended substrate. A detailed description of that measurement method can be found in [36], and the resonator appearance was shown in Figure A2. As a result, the capacity and quality factor of capacitors were obtained.

The capacity and quality factor of the planar capacitors based on ferroelectric film were then recalculated into dielectric permittivity and tangent loss of the ferroelectric film with the use of the partial capacity method, proposed by Vendik [37].

The measured capacitors’ parameters and the calculated parameters of ferroelectric film for the investigated samples, samples 2 and 3, can be found in Table A1 and Table A2, respectively.

**Table A1:** Planar capacitors investigations for Sample 2.

Sample	*C*, pF	*Q*	ε	tanδ
1	0.283	79	335	0.017
2	0.272	58	318	0.024
3	0.278	39	328	0.035
4	0.274	61	322	0.023
5	0.279	42	330	0.033
6	0.274	48	321	0.029
7	0.281	39	332	0.035
8	0.282	75	334	0.018
9	0.288	50	344	0.027
10	0.276	63	325	0.022
			329 ± 4	0.026 ± 0.003

**Table A2:** Planar capacitors investigations for Sample 3.

Sample	*C*, pF	*Q*	ε	tanδ
1	0.251	370	285	0.0038
2	0.242	400	271	0.0036
3	0.245	450	275	0.0032
4	0.234	340	258	0.0043
5	0.237	350	262	0.0041
6	0.238	365	263	0.0040
7	0.244	430	273	0.0033
8	0.238	320	263	0.0046
9	0.242	350	270	0.0042
10	0.230	390	251	0.0038
			267 ± 5	0.004 ± 0.0002
